# Evaluation of Starch–Protein Interactions as a Function of pH

**DOI:** 10.3390/foods8050155

**Published:** 2019-05-07

**Authors:** Ángela Bravo-Núñez, Raquel Garzón, Cristina M. Rosell, Manuel Gómez

**Affiliations:** 1 Food Technology Area, College of Agricultural Engineering, University of Valladolid, 34071 Palencia, Spain; pallares@iaf.uva.es; 2 Institute of Agrochemistry and Food Technology (IATA-CSIC), C/Agustin Escardino 7, 46980 Paterna, Spain; ragarllo@gmail.com (R.G.); crosell@iata.csic.es (C.M.R.)

**Keywords:** starch, pH, proteins, pasting properties, texture

## Abstract

Protein–starch gels are becoming more common in food processing when looking for enriched foods. However, processing conditions scarcely are considered when producing those gels. The aim of this research was to study the effect of processing pH (4.5, 6.0, and 7.5) on the hydration and pasting properties, gel microstructure, and texture of corn starchy gels made with four different proteins (pea, rice, egg albumin, and whey) at a ratio of 1:1 starch/protein and a solid content of 12.28%. The water binding capacity of the starch–protein mixtures was positively influenced by low solubility of the protein used. Acidic pH decreased the apparent peak viscosity of both starch and starch–protein mixtures, with the exception of starch–albumin blends, which increased it. The gels’ microstructure showed that the uniformity of the protein-enriched gels was dependent on protein type and pH, leading to diverse hardness. In general, the starchy gels containing animal proteins (albumin and whey) were more affected by pH than those obtained with vegetal proteins (pea and rice). Therefore, processing pH might be an advisable method to modify the functionality of starch–protein gels.

## 1. Introduction

The increasing demand for high-protein foods has prompted the development of a number of foods, particularly those dealing with high-protein starchy products [1], in which proteins can have either a technological or nutritional role. Numerous studies have been focused on combining vegetal or animal proteins with starchy matrixes leading to different gels. In fact, within the vegetal proteins, starch interactions with proteins from legume [2,3,4], peanut [5], and wheat proteins [6,7] stand out. Concerning starch–animal protein interactions, research mostly has been focused on the interactions of different starches with milk proteins [2,8,9,10,11,12]. On top of this, Bravo-Núñez and Gómez [13] focused on the interactions of milk, albumin, and collagen proteins with corn flour. 

Proteins are known as structure- and texture-macromolecular modifiers in foods, where multiple interactions with the rest of the biopolymers can take place [14], but their impact is dependent on both their intrinsic characteristics and media conditions. Consequently, to develop protein–starch products maximizing the interaction between both biopolymers, it is advisable to know the functional properties of each protein and their performance when blended with starch to understand their potential behaviors in real food matrixes. It is well known that each protein has a unique three-dimensional structure, resulting from the attractive and repulsive interactions emanating from the secondary and tertiary structures of the protein molecules [15] and that many physicochemical properties are derived from those structures. Nevertheless, extrinsic factors, like process pH, affect the electrostatic charge of the proteins, modifying their behavior in food processing depending on the used pH [16]. Since starchy food products embrace a wide range of pHs, from pHs in the range of 4.0–5.0 in sourdough breads [17] to pHs in the range of 6.0–7.5 in the case of cakes [18], the effect of process pH will determine the starch–protein interactions and their behaviors should be considered for each scenario. Despite the large range of conditions in food processing, to the best of our knowledge, the pH impact on gels has been only considered on the interaction of corn starch and whey protein [12] and the mixture of starch and lentil protein [3]. Therefore, understanding the starch–protein interactions during food processing remains very much unexplored, considering the variety of proteins commonly used as ingredients in the food industry. 

The aim of the present study was to understand the starch–protein interactions over a range of pH conditions that are faced during processing of starchy foods. With that purpose, mixtures of corn starch, one of the most used starches worldwide, with vegetal (pea and rice) and animal (albumin and whey) protein, at a ratio of 1:1 starch/protein, were subjected to different pHs (4.5, 6.0, and 7.5) and changes in hydration, apparent viscosity of paste and gels, textural properties, and microstructure were evaluated. The mixtures were dry-blended before analysis. The pHs were chosen to cover the pH range that can be found in starchy products.

## 2. Materials and Methods

### 2.1. Materials

Corn starch (Roquette, Lestern, France), rice protein (≥79% protein) (Remypro N80+, Remy Industries, Leuven-Wijgmaal, Belgium), pea protein (≥84% protein) (Nutralys F85F, Roquette, Lestrem, France), albumin (≥80% protein) (Albumin powder Specialist, Occhiobello, Italy), and whey protein (≥88% protein) (Provolon 295, Glanbia, Ballyragget, Ireland) were used. Acetate buffers (0.05–0.10 M) were prepared with glacial acetic acid and sodium acetate for pHs up to 5.0 and phosphate buffers (0.05–0.10 M) with sodium dihydrogen phosphate anhydrous and disodium hydrogen phosphate anhydrous for pHs over 5.0. All chemicals were purchased from PanReac AppliChem (Castellar del Vallès, Barcelona, Spain). 

### 2.2. Methods

#### 2.2.1. Protein Turbidity and Water Binding Capacity of the Starch, Proteins, and Starch–Protein Mixtures

The protein turbidity (OD 500) was determined in the pH range of 2.0–9.0 using 0.05 M acetate buffer for pHs 2.0–5.0 and 0.05 M phosphate buffer for pHs between 6.0 and 9.0. HCl or NaOH were used to adjust pH. Protein samples (0.2% *w*/*v*) were suspended in the different pH buffers and mixed for 10 min. Then, turbidity was measured in a spectrophotometer (UV mini-1240, Shimadzu Corporation, Kyoto, Japan) at 500 nm [19]. Four replicates for each measurement were carried out.

Water binding capacity (WBC) was measured as described in method 56.30 [20] with the modifications of Bravo-Núñez and Gómez [13] and 25 g of buffer (0.1 M) was added to 1.25 g (±0.1 g) of sample, mixed vigorously, and centrifuged at 2000 rpm for 10 min. The hydrated solid was weighed after removing excess water and the results were expressed as grams of retained water per grams of powder sample. WBC was analyzed in duplicate.

#### 2.2.2. Pasting Properties

Pasting properties were determined using a Rapid Visco Analyser (Model RVA-4C, Newport Scientific Pty. Ltd., Warriewood, Australia). For each sample, 3.5 g (±0.1 g) was dispersed in 25 g (±0.1 g) of buffer (0.1 M) following the indications of Martinez et al. [21]. The obtained slurry was then exposed to the general pasting method 61–02.01 [20]. The slurry was held at 50 °C for 1 min, then heated to 95 °C and held at that temperature for 2 min 30 s. It was subsequently cooled to 50 °C and held at that temperature for 4 min. All measurements were performed in duplicate. Pasting properties were performed in duplicate. 

Starchy gels obtained after the Rapid Visco Analyser cycle were poured into cylindrical containers (180 mm of height and internal diameter of 350 mm) and left to cool down at room temperature for one hour. Containers were stored at 4 °C for 24 h to achieve gel stabilization and further analysis was carried out. 

#### 2.2.3. Gel Microstructure

The pH effect on the gel structures obtained from the pasting test was recorded using a fluorescence microscope Nikon eclipse 90i (Nikon Corp., Tokyo, Japan). A portion of each gels were stained with 15 µL of Nile blue (Sigma-Aldrich Chemie Gmbh, Munich, Germany) solution (0.1 g/100 mL water) and then gels were observed at 16× magnification.

#### 2.2.4. Gel Texture

Texture measurements of the gels in the cylindrical containers described above (180 mm in height with an internal diameter of 350 mm) were performed at room temperature (≈25 °C) with a TA.XT2i Texture Analyzer (Stable Micro Systems Ltd., Surrey, UK) using a 5 kg load as the calibration force. One compression cycle was applied using a 6 mm diameter cylindrical probe (SMS P/6) at a constant crosshead velocity of 0.5 mms^−1^ to a sample depth of 10 mm. Hardness (N) was defined as the first peak force observed during the compression. All measurements were performed in duplicate.

#### 2.2.5. Statistical Analysis

Analysis of variance was used to study the differences between the characteristics of the mixtures according to the percentage and type of both flour and protein. Fisher’s least significant difference test was used to describe means with 95% confidence. The analysis was performed using Statgraphics Centurion XVII software (Statpoint Technologies, Warrenton, VA, USA).

## 3. Results and Discussion

### 3.1. Protein Solubility and Water Binding Capacity of the Mixtures

**Figure 1:**
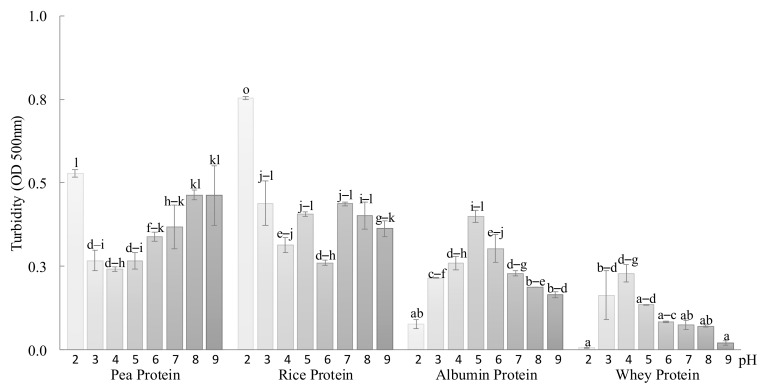
Turbidity (OD 500 nm) of each protein at different pHs. Columns with different letters differ significantly (*p* < 0.05).

Protein turbidity in the range of pH 2.0–9.0 is shown in Figure 1. Turbidity is inversely correlated with solubility, as insoluble mixtures have high turbidity due to light scattering by insoluble particles [22]. Although the proteins evaluated were not pure proteins, the assessment of turbidity was carried out to identify the pI, because all samples had a protein content higher than 79%. Animal proteins showed a solubility decrease at acidic pHs, specifically at pH 5.0 in the case of albumin and 4.0 in the case of whey protein. 

The reduction of solubility was close to the isoelectric points (pI) already described; 4.5 for albumin [23] and 5.0 for whey protein [24], which are in agreement with the results of Machado et al. [25] and Pelegrine and Gasparetto [26], who studied albumin and whey protein solubility as a function of pH, respectively. At the pI, the electrostatic forces between the protein molecules were minimum, reducing water–protein interactions. This is a favorable condition for the approximation and aggregation of protein molecules, leading to a low solubility [27]. On the contrary, vegetal proteins presented a different behavior. Regarding pea protein, with a pI of 4.5 [28], it showed its highest solubility between pH 3.0 and 5.0, close to its pI. When the pH was increased, the solubility decreased and the same was observed when the pH was decreased, especially at pH 2.0. Conversely, rice protein presented an unclear pattern, but higher solubility values were observed at pHs 4.0 and 6.0. The pI of rice protein was reported to be between 4.0 and 4.5 [29]. Although it is widely accepted that protein solubility decreases around the pI due to protein aggregation, which was observed with the animal protein, Damodaran [15] previously reported that some proteins present high solubility near the pI. This author hypothesized that the properties of the protein surface and the thermodynamics of its interaction with the surrounding solvent are more important for its solubility characteristics than the global average hydrophobicity and/or the charge frequency of the protein as a whole, which could justify the results obtained with the vegetal proteins. 

The WBC of the individual proteins and the blended starch–proteins are shown in Table 1. Starch WBC was not influenced by the pH of the media, while tested animal proteins showed null WBC capacity, regardless of the pH. It is known that acidic conditions can degrade starch and modify the WBC [30], but a certain time in the acidic media is needed (usually WBC is completely lost after 24 h), which did not happen in this case. 

Regarding the vegetal proteins, the pea protein significantly increased its WBC when the pH was increased, while the rice protein only showed a significantly higher WBC at pH 7.5 with respect to the other two tested pHs. The increase of the WBC at a higher pH might be related to the solubility decrease at alkaline pHs of these two proteins (Figure 1), in agreement with Shin et al. [31] and Bravo-Núñez and Gómez [13], who previously reported a relationship between lower solubility and higher WBC capacity. Although sole albumin and whey protein had null WBC, starch–whey and starch–albumin mixtures had WBC. Mixtures with one or the other protein showed a higher WBC at pH 4.5, near the pI of both proteins. In fact, the starch–whey mixture only showed the ability to retain water (WBC) at this pH. This again can also be related with protein solubility, as both proteins decreased in solubility when closer to the pI. 

The starch–pea protein mixture showed the same trend as the sole protein, presenting a higher WBC with a higher pH, while the starch–rice protein mixtures did not present a pH dependence. Both mixtures presented significantly lower WBC values than their counterpart proteins at each pH. This seems logical, as the WBC of the vegetal proteins was higher than the WBC of starch, in opposition to what occurs with animal proteins. The fact that the starch–rice protein mixtures did not present a pH dependence can be related to that fact that the differences between the WBC of rice protein at the different tested pHs were much smaller than the differences between WBC of pea proteins. On account of this, it seems reasonable to believe that the differences induced by the pH in the rice protein are covered up by starch when in mixture form, while differences for pea protein are not. 

### 3.2. Pasting Properties

**Figure 2:**
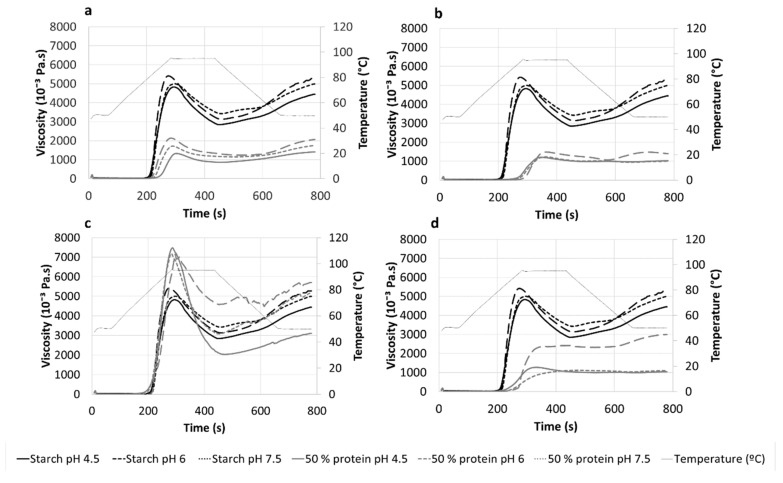
Effect of pH on the apparent viscosity plots of starch–protein mixtures (1:1), compared to the corn starch behavior. (**a**) Pea, (**b**) rice, (**c**) egg albumin, and (**d**) whey proteins. Legends: Starch viscosity (black lines), starch–protein viscosity (grey lines). pH 4.5 (continuous line), pH 6.0 (discontinuous line), pH 7.5 (dotted line).

Pasting properties of corn starch and starch–protein mixtures are presented in Figure 2. The starch displayed a lower viscosity profile at a lower pH, in agreement with the existing literature [30]. This decrease was attributed to the hydrolysis of amorphous regions and the production of low molecular weight dextrins that tended to dissolve rather than swell when heating in water [32,33]. In addition, a slightly upward shift of pasting temperature was observed, from 78.25 ± 0.00 °C when the pH was 7.5, to 79.48 ± 0.03 °C when the pH was 4.5. It was previously reported that high starch concentration leads to low pasting temperatures, while higher concentration of mono- and oligo-saccharides result to an upward shift of this temperature [34]. This can also be the underpinning reason for our results, as acidic conditions favor hydrolysis of the amorphous parts within starch granules and subsequently the increase of low molecular weight dextrins in our slurries. Another interpretation is that the preferential hydrolysis of the amorphous regions attenuates the destabilizing effect of swelling in these regions on the melting of the crystallites [35,36], retarding starch gelatinization. 

Substitution of 50% of the starch by either pea, rice, or whey proteins resulted in an overall decrease in the apparent viscosity profile, which might be ascribed to the reduction or dilution of starch content. Nevertheless, the apparent viscosity decrease was larger than that expected due to starch dilution, suggesting that starch–protein interactions limited the starch swelling and the changes associated to gelatinization and gelling. These trends agree with the results of Bravo-Núñez and Gómez [13], who considered the interactions of different animal proteins with corn flour without modifying the pH, and Onwulata et al. [9], who focused on the interactions between whey protein and different starches. Within each starch–protein mixture, lower viscosity profiles were displayed when the pH was, following the trend observed for the sole starch. From our results, it seems that the influence of pH on starch pasting properties is higher than its influence on proteins, unlike what we observed for the WBC. Nevertheless, the impact of pH on the apparent viscosity of whey protein–starch is noteworthy. Viscosity of whey–starch mixtures (Figure 2d) at pH 7.5 was significantly higher during heating and cooling than at pHs 6.0 and 4.5. Although similar solubility in the pH range of 6 to 8 was observed (Figure 1), Pelegrine and Gasparetto [26] observed that, upon heating, the solubility of whey protein at pH 7.8 was decreased. Since this pH is close to pH 7.5, this indicates that thermal protein denaturation at this pH may be occurring, which could justify our viscosity increase at pH 7.5. Anyhow, the presence of any of these three proteins (pea, rice, or whey protein), regardless of the pH, resulted in an upward shift in temperature/time of the pasting temperature with respect to the starch at the corresponding pH. Rasper [37] reported that the pasting temperature is affected by the starch concentration, which was half in our mixtures than in the sole starch sample. On top of that, Noisuwan et al. [10] reported that proteins may be adsorbed onto the starch granules, restricting the diffusion of water into the starch granules during pasting, which could delay the pasting temperature. 

The mixtures of corn starch and albumin showed a completely different pattern, increasing the peak viscosity with respect to the one obtained with starch, regardless of the pH and in spite of starch dilution. This particular behavior of the albumin protein was previously reported by Bravo-Núñez and Gómez [13]. Although that study did not consider the influence of pH, an increase in the apparent viscosity of starch was induced by this protein. This behavior was associated with the coagulation of albumin proteins [38], which occurs in the same temperature range as starch gelatinization [39]. In addition, peak viscosity was higher when the pH was lower (7362 ± 163, 7164 ± 78, and 7158 ± 54 10^−3^ Pa·s for pH 4.5, 6, and 7.5 respectively), in opposition to what was observed with the other tested mixtures. Van Kleef [40] reported higher storage moduli in albumin gels at a low pH (5.0) than at a high pH (10.0), owing to the formation of an aggregated particle network at a low pH, in which the compact rigid protein molecules strongly interact (high levels of both inter- and intramolecular protein–protein interactions), favoring their coagulation and high viscosity. This assumption was also supported by the solubility results, because albumin showed the lowest solubility at pH 5.0. Nevertheless, a higher final viscosity was observed for the starch–albumin gels at higher pHs (6 and 7.5) than at pH 4.5, which agrees with the trend observed with the other starch–protein mixtures, even showing a lower final viscosity than the individual starch in the case of pH 4.5. Again, this could be related to the formation of compact rigid albumin aggregates at a low pH (probably due to the low solubility of the protein at pH 5), resulting in low values of deformation during breakage and therefore in a much weaker gel [40].

### 3.3. Gel Microstructure

**Figure 3:**
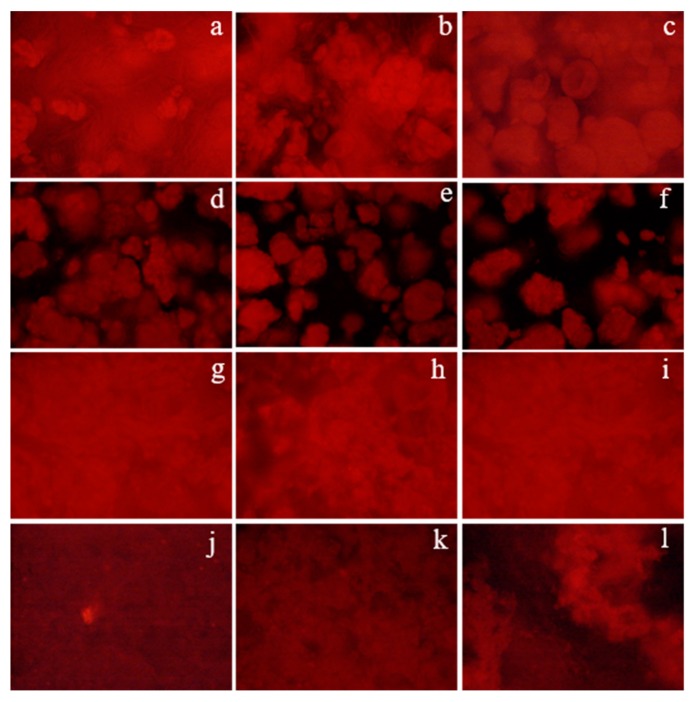
Optical microscope images (×16) of different starch–proteins gel (1:1) at different pHs (4.5, 6.0, 7.5). (**a**–**c**): Starch–pea protein; (**d**–**f**): Starch–rice protein; (**g**–**i**): Starch–egg albumin; (**j**–**l**): Starch–whey protein at pHs 4.5, 6.0, and 7.5, respectively.

The gels containing different proteins were stained and observed under fluorescence (Figure 3). Differences among gels were readily evident, adopting different structure depending on the protein source. Gels were observed as homogeneous films, with the exception of gels containing rice proteins (Figure 3d–f), which were displayed as aggregates. Nevertheless, the gels’ uniformity was dependent on the pHs used for their preparation. In fact, gels containing pea protein or albumin exhibited slightly discontinuous gels at pH 6.0 (Figure 3b,h). In the case of whey protein, the gel structure was progressively lost when the pH was increased and dense structures were observed at pH 7.5 (Figure 3l). In the case of gels containing rice proteins, the gel structure could be only envisaged at pH 4.5 (Figure 3d), but very well-defined, compact structures were observed at higher pHs (Figure 3e,f). The temperature- and pH-induced gelation of the proteins have already been reported [41], since protein structure is pH-dependent, tending to form aggregates close to the isoelectric point (pI) due to the decrease of electrostatic repulsion forces that favors the hydrophobic interactions and, in consequence, the formation of particulate gels. The food industry very often uses protein isolates for enriching proteins, especially cereal-based goods, but commercial isolates contain a mixture of proteins rather than a purified protein.

The behavior of the gels obtained at different pHs can be related to the solubility of the protein isolates (Figure 1) that showed completely different patterns, as explained in 3.1. Therefore, the pH-induced aggregation of the commercial proteins and the pH differed from the one reported as pI for the individual proteins, since pI 4.5 has been reported for pea and ovalbumin proteins [23,28], pI 5.0 for whey protein [24], and the pI for rice has been described between 4.0 and 4.5 [29].

Considering the microstructure results of the gels, it seems that aggregates of albumin could be explained by protein interactions because aggregation in the gel was more visible at pH 6.0, close to the maximum turbidity (and therefore lower solubility) observed. However, when gels were made at pHs of 4.5 or 7.5, proteins were more integrated within the starch network. In fact, Villanueva et al. [42] obtained different gels by mixing different starches (rice, tapioca, and potato) with egg albumin and reducing the pH to 4.5. In the case of the gels containing whey and pea protein isolates, aggregate formation did not agree with the solubility pattern of the proteins, therefore starch chains might interfere with the protein aggregation, or these isolates were more prone to forming aggregates with starch polymers at specific pHs. In some starches, such as corn, potato, and rice, it has been described that, at acidic pH levels, the resulting starchy gels had lower viscosity due to the higher corrosion of the amorphous region of the starch [43]; this fact could help the linkage between protein and starch-originating gels with a more integrated structure.

### 3.4. Gel Texture

The hardness of the gels from the starch and starch–protein mixtures is presented in Table 1. Our results showed no significant differences for starch gels obtained at different pHs, in opposition to the results of the RVA, where a lower pH resulted in a lower final viscosity. According to the literature, acidified starches resulted in softer gels because of the low number of high molecular weight amylose molecules due to hydrolysis [44]. Likely, the lack of differences in the present study can be related to the minor extent of the acid hydrolysis of starch.

Starch–rice mixtures did not reach a gel-like structure after 24 h of storage at 4 °C as microscope images revealed, thus the texture could not be assessed. In an ideal starch gel, the firmness will initially increase with time as a consequence of amylose change from an amorphous to a more orderly and crystalline state [4]. If this “ideal” gel also contains other components like proteins, they will most likely interfere in the reorder of amylose, resulting in a decrease of gel hardness or in a non-formed gel, as happened with our starch–rice protein gel.

Regarding the other mixtures, independently of the pH, the substitution of starch by any of the proteins resulted in a significant decrease in hardness compared to the starch gel, with the exception of starch–whey protein mixture at pH 7.5 that did not show a significant difference with the starch gel. This reduction seems logical due to starch having better gelling properties than protein. Moreover, it must be considered that, as stated in the previous paragraph, the position of the proteins inbetween swollen starch granules and leaching material is functioning as an inactive filler, changing the gels’ microstructure. This was previously reported by other authors like Ribotta et al. [4] with starch–soy protein blends and Yang et al. [45] with starch–dairy blends, although they did not consider the effect of pH. Concerning the effect of the pH, no significant differences were observed among the gels containing albumin or pea proteins. For starch–whey protein gels, a significant hardness increase was obtained at pH 7.5, which agrees the RVA results, where the final viscosity was notoriously higher in the range of pH 6–8 than at the two other pHs. As it can be observed in Figure 1, at pH 7.5, whey protein is more soluble than the other proteins, which may result in less interference of the protein within the starchy gel, minimizing hardness loss. The gel microstructure of starch–whey mixtures also supported this result. 

## 4. Conclusions

Gel properties of starch–protein mixtures can be modulated with the media pH, which should be taken into consideration when developing enriched starchy products (e.g., cookies, cakes, or breads) based on these kind of mixtures. The water binding capacity of the mixtures increased with the pH of lower protein solubility. Protein addition reduced the apparent viscosity of the starchy gels, except for the starch–albumin gels. Acidic pHs decreased the apparent viscosity during heating and cooling and increased the pasting temperature and again, starch–albumin gels showed the opposite performance regarding the pH.

Gel images confirmed that the gel microstructure was related to the pH, although different microstructures did not always lead to hardness changes. Only in the case of starch–whey protein gels were harder gels obtained at pH 7.5, with respect to the control. 

## Figures and Tables

**Table 1:** Hydration and hardness of corn starch and proteins individually and in corn starch–protein mixtures/gels.

	WBC			Hardness (N)		
	pH 4.5	pH 6.0	pH 7.5	pH 4.5	pH 6.0	pH 7.5
Starch	0.72 ± 0.00 ^d^	0.75 ± 0.01 ^d^	0.75 ± 0.02 ^d^	0.79 ± 0.10 ^cd^	1.00 ± 0.06 ^d^	0.77 ± 0.03 ^cd^
Pea protein	2.69 ± 0.16 ^ij^	3.37 ± 0.06 ^k^	4.09 ± 0.04 ^l^	nd	nd	nd
Rice protein	2.6 ± 0.04 ^hi^	2.52 ± 0.04 ^h^	2.75 ± 0.05 ^j^	nd	nd	nd
Egg albumin protein	nd	nd	nd	nd	nd	nd
Whey protein	nd	nd	nd	nd	nd	nd
Starch–pea protein	1.46 ± 0.00 ^e^	1.60 ± 0.01 ^f^	2.00 ± 0.03 ^g^	0.25 ± 0.03 ^a^	0.15 ± 0.00 ^a^	0.09 ± 0.00 ^a^
Starch–rice protein	1.48 ± 0.05 ^ef^	1.39 ± 0.01 ^e^	1.48 ± 0.03 ^ef^	nd	nd	nd
Starch–egg albumin protein	0.35 ± 0.07 ^c^	0.08 ± 0.03 ^ab^	0.14 ± 0.08 ^b^	0.34 ± 0.04 ^ab^	0.23 ± 0.01 ^a^	0.37 ± 0.03 ^ab^
Starch–whey protein	0.02 ± 0.00 ^a^	nd	nd	0.10 ± 0.02 ^a^	0.09 ± 0.00 ^a^	0.55 ± 0.44 ^bc^

Water binding capacity (WBC). No data (nd). Values followed by the same letter within the same analysis (WBC or hardness) are not significantly different (*p* < 0.05).
